# An insight into role of amino acids as antioxidants via NRF2 activation

**DOI:** 10.1007/s00726-024-03384-8

**Published:** 2024-03-20

**Authors:** Melford C. Egbujor, Olugbemi T. Olaniyan, Chigbundu N. Emeruwa, Sarmistha Saha, Luciano Saso, Paolo Tucci

**Affiliations:** 1 Department of Chemistry, Federal University Otuoke, Otuoke, Bayelsa Nigeria; 2 Department of Physiology, Rhema University Nigeria, Aba, Abia Nigeria; 3 Department of Chemistry, Rhema University Nigeria, Aba, Abia Nigeria; 4 Department of Biotechnology, Institute of Applied Sciences and Humanities, GLA University, Mathura, 281406 India; 5 Department of Physiology and Pharmacology, Vittorio Erspamer, Sapienza University of Rome, 00161 Rome, Italy; 6 Department of Clinical and Experimental Medicine, University of Foggia, 71122 Foggia, Italy

**Keywords:** Amino acids, Oxidative stress, NRF2-KEAP1 signaling, Antioxidant, Anti-inflammatory

## Abstract

Oxidative stress can affect the protein, lipids, and DNA of the cells and thus, play a crucial role in several pathophysiological conditions. It has already been established that oxidative stress has a close association with inflammation via nuclear factor erythroid 2-related factor 2 (NRF2) signaling pathway. Amino acids are notably the building block of proteins and constitute the major class of nitrogen-containing natural products of medicinal importance. They exhibit a broad spectrum of biological activities, including the ability to activate NRF2, a transcription factor that regulates endogenous antioxidant responses. Moreover, amino acids may act as synergistic antioxidants as part of our dietary supplementations. This has aroused research interest in the NRF2-inducing activity of amino acids. Interestingly, amino acids' activation of NRF2-Kelch-like ECH-associated protein 1 (KEAP1) signaling pathway exerts therapeutic effects in several diseases. Therefore, the present review will discuss the relationship between different amino acids and activation of NRF2–KEAP1 signaling pathway pinning their anti-inflammatory and antioxidant properties. We also discussed amino acids formulations and their applications as therapeutics. This will broaden the prospect of the therapeutic applications of amino acids in a myriad of inflammation and oxidative stress-related diseases. This will provide an insight for designing and developing new chemical entities as NRF2 activators.

## Introduction

Amino acids are natural organic compounds with basic amino and acidic carboxyl groups. It is well established that amino acids are essential molecules that will be of notable therapeutic importance in a wide range of diseases if well harnessed. Owing to their broad biological activities and abundance in nature, they have attracted increasing research interest over the last few decades. It is supposed that the structural uniqueness, abundance, widely studied biosynthesis, and numerous pharmacological activities have repositioned them as molecules that may be of therapeutic value in uprising diseases, including inflammation and oxidative stress-mediated diseases. Over the years, oxidative stress has been shown to play a significant role in the pathogenesis of several diseases through pro-oxidant factors altering several enzymes, cellular structures, gene expression, and mitochondrial physiology (Egbujor et al. 2020b, 2023a). Numerous studies have indicated that KEAP1 has an intracellular sensing ability to act as a homodimer on the cell’s redox status in modulating the biological functions of the transcription factor NRF2 (Suzuki and Yamamoto 2015; Egbujor et al. 2023b). (Cuadrado et al. 2018) reported that NRF2 is a molecular target for protein–protein interaction within the cell for cytoprotective ability. The authors suggested that these interactions are essential for alteration in the expression of the transcription factor NRF2 which has been linked with systemic protection from several disease conditions like metabolic alterations, chronic inflammation, autoimmune, digestive, respiratory, cardiovascular, neurodegenerative diseases, and cancer. Also, (Cloer et al. 2019) reported that the NRF2–KEAP1 signaling pathway could be activated through protein–protein interaction to suppress cancer-associated risk factors such as xenobiotic efflux, cellular resistance to oxidative stress, metabolic reprogramming, and proliferation. The authors suggested that this mechanism may be targeted for cancer treatment. (Chen et al. 2015) revealed that in the endothelial cell, NRF2–KEAP1 signaling plays a significant role in modulating the redox homeostasis, thus preventing several cardio-metabolic diseases like atherosclerosis, hypertension, aging, and ischemia, through combination with antioxidant response elements (ARE) and subsequently promoting several antioxidant genes transcription. During oxidative stress or inflammation, NRF2 dissociates from KEAP1, resulting in nuclear transcription processes (Egbujor et al. 2023b). (Yamamoto et al. 2018) described the NRF2–KEAP1 system as a thiol-based biosensor regulating redox status in the cell. The NRF2–KEAP1 system is essential for regulating electrophilic stress and oxidative reaction within the cellular origin (Egbujor et al. 2021, 2022).

NRF2 is a transcription factor involved in the regulation of much protein–protein interactions like thioredoxin-glutathione antioxidant systems and unfolded protein responses, while KEAP1 display unique cysteine thiol-rich sensor activity. The two systems involve repression and inducibility mechanisms during stress in biological processes (Yamamoto et al. 2018). (Cheng et al. 2019) reported in their study that NRF2 activation is conferred with several functions such that in aging, its activation by protein FAM129B may lead to suppression of oxidative stress, and in cancer, such activation may advance the progression and metastasis of the disease through ubiquitination via ETGE and DLG motifs. Also, in a similar work, (Zhao et al. 2021a) explored the NRF2-mediated antioxidant effects of casein (a rich source of amino acids) derived antioxidant peptides. They discovered that the casein peptides exhibited significant antioxidant activity through the activation of NRF2 and mRNA gene expression of superoxide dismutase (SOD), glutathione peroxidase (GPx), and catalase (CAT). They also noted that the peptides could reduce the expression of KEAP1, malondialdehyde, and reactive oxygen species, thus, suggesting that casein peptides could be very useful in the treatment of cancer and many other diseases. This review focuses on the role of amino acids in NRF2 activation, their NRF2-mediated antioxidant activities, and their therapeutic effects in various diseases.

### Amino acids as NRF2 activators

Amino acids play a crucial role in the activation of the NRF2 signaling pathway. Studies have revealed that several amino acids, such as glutamine, glutamate, and aspartate, can stimulate the activity of NRF2 in the cell. The NRF2 controls glutamine as a nitrogen donor in the TCA cycle, thus modulating glutamine uptake and metabolism. Also, NRF2 participates in the regulation of important protein expression called ATF4, needed in the regulation of asparagine synthesis and glutamine SLC1A5 transporter for the overproduction of glutamine and asparagine. The NRF2 target gene in the cell is glutaminase responsible for the glutamine metabolism to glutamate (Mitsuishi et al. 2012). (Gacesa et al. 2018) reported that Mycosporine-like amino acids are potent activators of the NRF2–KEAP1 signaling pathway. Mycosporine-like amino acids are metabolites such as shinorine and porphyra-334 and are derived from marine algae and cyanobacteria with intense ultra-violet ray-absorbing properties. The authors confirm the ability of these amino acids to bind with the NRF2–KEAP1 signaling pathway using thermal shift assays and fluorescence polarization techniques. Their study suggested that Mycosporine-like amino acids can increase transcriptional regulation and promote antioxidant capacity in aging and multiple degenerative dysfunctions. Furthermore, several amino acids, especially cysteine, act as a precursor in the synthesis of antioxidants and help to modulate NRF2 activity by a direct interaction (Courtney-Martin and Pencharz 2016; He et al. 2018). Similarly, NRF2 influences the availability of amino acids through ATF4 expression, which modulates the de novo biosynthesis of amino acids and the transcription of LAT 1 and ASCT 2, which are amino acid transporters (Wang et al. 2011, 2013). The intracellular concentrations of several amino acids are controlled by NRF2 (Mitsuishi et al. 2012; Hayes and Dinkova-Kostova 2014). In summary, NRF2 and amino acids are interdependent. Amino acids influence the activation of the NRF2 pathway, which controls the availability of amino acids via de novo biosynthesis. However, the interactions between the two are quite complex and depend on the nature and number of amino acids, the cellular context, and other cellular components (DeNicola et al. 2015; Gwinn et al. 2018; He et al. 2018; Guo et al. 2019).

Although the mechanisms by which amino acids activate NRF2 have not been fully elucidated, however, the available data suggest four possible mechanisms. The most popular among these mechanisms is the modulation of KEAP1, a negative regulator of NRF2. This involves the direct interaction of some amino acids, such as cysteine and homocysteine, with KEAP1, which allows NRF2 to translocate to the nucleus, and activate its target enzymes and genes (Bryan et al. 2013; Tebay et al. 2015; Panieri et al. 2020). Thus, these amino acids activate NRF2 by facilitating its nuclear translocation from the cytoplasm to the nucleus where it binds to anti-oxidant response element (ARE) in the promoter region of target genes and promotes their transcription. This process helps regulate antioxidant enzymes, and phase II detoxifying enzymes which results in cellular protection against oxidative stress and cellular damage (Bryan et al. 2013; Tebay et al. 2015; Panieri et al. 2020). Secondly, the activation of the PI3K/AKT signaling pathway has been found relevant to the activation of NRF2 by amino acids. Some of the amino acids activate the PI3K/AKT signaling pathway, which consequently leads to the activation of NRF2 (Zhang et al. 2013; Long et al. 2021). Thirdly, some amino acids induce oxidative stress, which in turn results in the activation of NRF2 (Elisia et al. 2011; Xu et al. 2018). Fourthly, some amino acids activate NRF2 by modulating gene expression, including the NRF2 gene, which in turn leads to NRF2 activation (Fafournoux et al. 2000; Kimball and Jefferson 2004). Here, we will discuss the NRF2-inducing activity of amino acids based on their classes as essential and non-essential amino acids.

### Role of essential amino acids in NRF2 activation

It is well established that humans cannot synthesize certain amino acids, so they must be sourced highly to meet the body's nutritional requirements. Thus, this class of amino acids is considered essential and indispensable. Many of these amino acids have been found to exhibit strong anti-oxidative capacity (Xu et al. 2017) and anti-inflammatory activities (Liu et al. 2022). Furthermore, some essential amino acids have been reported to exert activities via the activation of the NRF2 signaling pathway (Gan et al. 2014; Wu et al. 2020; Huang et al. 2021). These NRF2-activating essential amino acids include histidine, isoleucine, methionine, leucine, lysine, threonine, phenylalanine, valine, and tryptophan.

### Histidine

Alpha-amino acid histidine has an imidazole ring (**1**) (Table 1). It makes up most frequently the active site of protein enzymes (Ingle 2011). It is essential for both infants and adults (Kopple and Swendseid 1975). The first step in the production of histidine is the conversion of ribose-5-phosphate to 5-phosphoribosyl-1-pyrophosphate (PRPP). The enzyme PRPP synthetase is responsible for catalyzing this reaction. The histidine biosynthesis pathway is a sequence of enzymatic processes that is involved in the production of histidine in both bacteria and plants. Usually, the following important enzymes are part of the pathway: IPTG (imidazoleglycerol-phosphate) catalyzed by synthase, is formed when PRPP and l-glutamine condense. Phosphate of histidine (HOL-P) HOL-P is dephosphorylated by phosphatase, an enzyme that catalyzes the production of histidinol. Histidine is produced by a reduction process when histidine is oxidized to histidinal by the enzyme histidinol dehydrogenase. The final product, histidine, can control its own production since the histidine biosynthesis pathway is frequently susceptible to feedback inhibition (Alifano et al. 1996). Histidine’s ability to activate NRF2 has been well investigated in grass carp (*Ctenopharyngodon idella*). Histidine supplementation prevents Cu-induced oxidative damage by restoring Cu-mediated decreases in GPx and SOD levels by upregulating NRF2 mRNA expression levels in fish intestines (Jiang et al. 2016b). (Wu et al. 2020) reported that lack of dietary histidine significantly decreased nutrient contents in the fillet of on-growing grass carp and down-regulated NRF2 signaling, up-regulated KEAP1 gene expression, and total and nuclear NRF2 protein levels. The histidine supplementation reverses these effects and increases antioxidant enzyme’s mRNA levels by the upregulation of NRF2 mRNA level. The long-term effects of dietary  histidine are not examined in these investigations, nor were the detailed underlying mechanisms of how dietary  histidine influences fish flesh quality and antioxidant capacity. Histidine’s role in the NRF2-inducing activity and its effects on the growth efficiency and general antioxidant capacity of various fish species has been demonstrated in other investigations. For instance, dietary histidine significantly elevated the gene transcriptions and antioxidant enzyme activities in the head kidney via NRF2 activation and enhanced growth performance in juvenile hybrid grouper (*Epinephelus fuscoguttatus* with *Epinephelus lanceolatus*) that are heavily farmed (Taj et al. 2022). Histidine supplementation also improves intestinal antioxidant capacity by increasing the mRNA levels of CAT, SOD, and GP_x_ via the upregulation of NRF2 expression level in largemouth bass (*Micropterus salmoides*) (Liang et al. 2022).

**Table 1:** NRF2 activation by essential amino acids

S/N	Molecular structure	Biological activity	Effective concentration	Study model	NRF2 target genes	Disease of interest	Reference
1	Histidine	Antioxidant	3.7–12.2 g/kg	Grass carp intestine	SOD GPx	Oxidative stress	(Jiang et al. 2016b)
		Antioxidant	3.7–12.2 g/kg	Grass carp	SOD CAT GPx	Histidine deficiency	(Wu et al. 2020)
		Antioxidant	9.2 g/kg 21.08 g/kg	Hybrid groupers	TOR GHR1	Stunted growth Oxidative stress	(Taj et al. 2022)
		Antioxidant	0.89–1.67%	Largemouth bass	SOD CAT GPx	Inflammation	(Liang et al. 2022)
2	Isoleucine	Antioxidant	6.6–12.5 g/kg	Grass carp	CuZn-SOD GPx	Flesh quality loss	(Gan et al. 2014)
		Antioxidant	12.36–14.9 g/kg	Hybrid bagrid catfish	CuZn-SOD CAT GPx	Flesh quality loss	(Jiang et al. 2021)
		Antioxidant	9.3 g/kg	Grass carp	CAT GPx SOD	Oxidative stress	(Feng et al. 2017a)
3	Leucine	Antioxidant	1.40% 1.56%	Juvenile blunt snout bream	CAT SOD GPx HO-1	Oxidative stress	(Liang et al. 2018a)
		Antioxidant	12.8 g/kg	Grass carp	SOD CAT GPx	Flesh quality loss	(Deng et al. 2016)
		Antioxidant	0.25%	Piglets	SOD CAT GPx	Oxidative stress	(Chen et al. 2019)
		Antioxidant anti-inflammatory	2.77%	Juvenile golden pompano	SOD GPx	Oxidative stress Anti-inflammatory	(Zhou et al. 2020)
4	Lysine	Antioxidant	2.44%	Grass carp	CAT GPx	Oxidative stress	(Huang et al. 2021)
5	Methionine	Antioxidant	21.50 mg	Growing rats	SOD CAT GPx	Oxidative stress	(Wang et al. 2019a)
		Antioxidant	21.2–53.0 mg/g	Rats	CAT SOD HO-1	Oxidative stress	(Li et al. 2020)
		Antioxidant	Deprivation	HEK293 Cells	HO-1	Oxidative stress	(Hensen et al. 2013)
		Antioxidant	0.1–5.0 mg/ml	SH-SY5Y Cells	SOD CAT	PD	(Catanesi et al. 2021)
		Antioxidant	0.18 mg/dL	Dairy cows	NQO1 GPx GCLM GCLC	Oxidative stress	(Han et al. 2018)
		Antioxidant	4.0 g/kg	Laying duck breeders	HO-1 GPx	Oxidative stress	(Ruan et al. 2018)
6	Phenylalanine	Antioxidant	9.1 g/kg	Grass carp	SOD GPx	Oxidative stress	(Feng et al. 2017b)
7	Threonine	Antioxidant	13.77 g/kg	Hybrid catfish	CAT GPx	Growth retardation	(Zhao et al. 2020)
8	Tryptophan	Antioxidant	1250 mg/kg	Mice	NQO1, HO-1	Hepatitis	(Kimura and Watanabe 2016)
		Antioxidant	3.81–3.89 g/kg	Grass carp	SOD GPx	Oxidative stress	(Wen et al. 2014)
		Antioxidant	3.81 g/kg	Grass carp	SOD CAT GPx	Oxidative stress	(Jiang et al. 2016c)
9	Valine	Antioxidant	4.3–19.1 g/kg	Grass carp	CAT SOD GPx	Oxidative stress	(Luo et al. 2017)
		Antioxidant	1.21–1.94%	Juvenile hybrid grouper	GPx	Growth deficiency	(Zhou et al. 2021)
10	Taurine	Antioxidant	10–80 mM	Mouse spermatocytes (GC-2 Cells)	HO-1	Oxidative stress	(Yang et al. 2017)
		Antioxidant	2% w/v	Diabetic rat	HO-1	Diabetic neuropathy	(Agca et al. 2014)

### Isoleucine

Isoleucine (**2**) (Table 1) is a branched-chain essential amino acid. Its biosynthesis involves a series of enzymatic reactions both in bacteria and plants. The first step in the biosynthesis of isoleucine biosynthesis involves the enzyme acetolactate synthase. The condensation of two pyruvate molecules to produce acetolactate is catalyzed by this enzyme. Next, the enzyme acetohydroxy acid isomeroreductase transforms acetolactate into 2,3-dihydroxy-isovalerate. α-Ketoisovalerate is produced when dihydroxy acid dehydratase dehydrates dihydroxy-isovalerate. A transaminase enzyme catalyzes the transfer of an amino group from glutamate to α-ketoisovalerate thereby producing isoleucine and α-ketobutyrate (Nelson et al. 2021). The PRR and NRF2-ARE signaling pathways, among others, are activated by isoleucine (Gan et al. 2014; Mao et al. 2018). The optimum isoleucine supplementation raises Cu/Zn-SOD and GPx levels and activates NRF2. Additionally, its absence or overproduction results in grass carp flesh losing quality (Gan et al. 2014). These observations are confirmed by Jiang et al. (Jiang et al. 2021) in hybrid bagrid catfish (*Pelteobagrus vachelli* × *Leiocassis longirostris*). Finally, adequate isoleucine supplementation protects the grass carp gill’s structural integrity and strengthens antioxidant defenses by activating NRF2 (Feng et al. 2017a).

### Leucine

Leucine (**3**) (Table 1) is a branched amino acid present in almost all proteins. It is a non-polar aliphatic α-amino acid that exhibits notable antioxidant activities (Chen et al. 2019). Humans rely on food as a source of leucine, while microbes and plants produce leucine from pyruvic acid with the aid of several enzymes. The first step is the catalysis of the condensation of two pyruvate molecules to generate 2-acetolactate by the enzyme acetolactate synthase. The enzyme acetohydroxy acid isomeroreductase then transforms acetolactate into 2,3-dihydroxy-isovalerate. α-Ketoisovalerate is formed when dihydroxy acid dehydratase dehydrates dihydroxy-isovalerate. The conversion of glutamate to α-ketoisovalerate, which results in the production of leucine and α-ketoisocaproate, is catalyzed by the transaminase enzyme (Nelson et al. 2021). (Liang et al. 2018a) reported that dietary leucine activates the NRF2 signaling pathway, elevates the expression of antioxidant genes and enzymes, and improves the antioxidant defenses, growth, and immunity of juvenile blunt snout bream (*Megalobrama amblycephala*). Optimum dietary leucine improves the flesh quality of grass carp by promoting its antioxidant defenses via the activation of NRF2 signaling molecule and elevation of its target enzymes (Deng et al. 2016) and enhanced intestinal antioxidant status, immunity, and growth in juvenile golden pompano (*Trachinotus ovatus*) (Zhou et al. 2020). About 0.25% dietary leucine supplementation increased the antioxidant activity and expression of the mRNA levels of NRF2 and its mitochondrial-related genes in the muscle and liver of piglets (Chen et al. 2019).

### Lysine

Lysine (**4**) (Table 1) is an essential α-amino acid that is a precursor to many proteins. The first step in its biosynthetic route is the conversion of aspartate to β-aspartyl phosphate, which is made possible by the enzyme aspartokinase. Next, aspartate-semialdehyde dehydrogenase catalyzes a process that transforms β-aspartyl phosphate into β-aspartyl-4-semialdehyde. D-aspartyl-4-semialdehyde is further converted by dihydrodipicolinate synthase into dihydrodipicolinate. Tetrahydrodipicolinate is produced when dihydrodipicolinate reductase breaks down dihydrodipicolinate. Next, tetrahydrodipicolinate undergoes acetylation, which is mediated by tetrahydrodipicolinate acetyltransferase to yield *N*-acetyl-tetrahydrodipicolinate. Catalyzed by *N*-acetyl-tetrahydrodipicolinate synthase, *N*-acetyl-tetrahydrodipicolinate is changed into *N*-succinyl-l-2-amino-6-oxopimelate. *N*-succinyl-l-2-amino-6-oxopimelate hydrolyase then produces l-2,6-diaminopimelate by hydrolyzing *N*-succinyl-l-2-amino-6-oxopimelate. Catalyzed by diaminopimelate epimerase, l-2,6-diaminopimelate undergoes epimerization to create meso-diaminopimelate. Finally, diaminopimelate is converted by diaminopimelate dehydrogenase into lysine (Miyazaki et al. 2004; Xu et al. 2006). Lysine exhibits antioxidant and anti-inflammatory activities (Cheng et al. 2020; Huang et al. 2021). Its anti-inflammatory action via micro-RNA-575/PTEN signaling has been linked to neuroprotection in mouse intracerebral hemorrhage injury (Cheng et al. 2020). Dietary lysine upregulates the mRNA expression levels of NRF2-dependent antioxidant genes such as CAT and GP_x_ in grass carp (Huang et al. 2021).

### Methionine

Methionine (**5**) (Table 1) is the precursor of some amino acids such as taurine and cysteine. The precursor for methionine biosynthesis is homocysteine, which is derived from methionine by transmethylation. Homocysteine receives a methyl group from methionine, which forms *S*-adenosylmethionine (SAM). The formation of *S*-adenosylmethionine (SAM) from methionine and ATP is catalyzed by methionine adenosyltransferase. SAM participates in a number of different cellular methylation processes as a methyl donor. To produce *S*-adenosylmethioninamine, *S*-adenosylmethionine is decarboxylated and *S*-adenosylmethionine decarboxylase is the catalyst for this process. The production of polyamines involves *S*-adenosylmethioninamine and 5'-methylthioadenosine (MTA) is a byproduct of this process. To regenerate methionine, the cell recycles MTA. MTA undergoes phosphorylation to generate 5'-methylthioadenosine-5'-phosphate (MTA-P) and subsequent reactions lead to the regeneration of methionine (Ferla and Patrick 2014). The chemical synthesis is achieved through acrolein, methanethiol, and cyanide reaction to afford the hydantoin (Elvers and Bellussi 2011). Methionine and its derivatives exhibit antioxidant and anti-inflammatory activities (Luo and Levine 2009; Egbujor and Okoro 2019; Sharma et al. 2019). l-methionine attenuates ROS-mediated oxidative stress by inducing endogenous antioxidant activity by activating the NRF2–ARE signaling pathway in growing rats (Wang et al. 2019a). Methionine’s availability also improves the endogenous antioxidant capacity of rice protein via the activation of NRF2–ARE signaling and stimulation of the methionine sulfoxide reductase (MSR) antioxidant system in both growing and adult rats (Li et al. 2020). Methionine deprivation renders heat shock factor 1 (HSF1) inactive, and HEK293 cells experience oxidative stress as a result (Hensen et al. 2013). Heat shock factor 1 (HSF1) is rendered inactive by methionine deprivation, and HEK293 cells experience oxidative stress as a result (Hensen et al. 2013). In Parkinson's disease, l-methionine prevents oxidative damage and mitochondrial dysfunction. It alters NRF2 signaling and prevents oxidative damage by 6-hydroxydopamine (6-OHDA) (Catanesi et al. 2021). Through the phosphorylation of NRF2 and its antioxidant enzymes, methionine has a favorable impact on antioxidant capacity in the mammary gland of dairy cows (Han et al. 2018). Methionine reduces the expression of hepatic NRF2 and associated genes, which enhances productive and reproductive performance in laying duck breeders (Ruan et al. 2018).

### Phenylalanine

Phenylalanine (**6**) (Table 1) is found in milk, meat, and eggs, and biosynthesis is achieved via the Shikimate pathway. The first step in the process is the conversion of erythrose-4-phosphate and phosphoenolpyruvate to shikimate, which is carried out by the enzyme 3-deoxy-D-arabino-heptulosonate-7-phosphate synthase (DAHPS). After this, a sequence of enzyme processes transform shikimate into chorismate. 5-enolpyruvylshikimate-3-phosphate synthase (EPSP synthase) is one of the important enzymes in this process. Chorismate mutase is the enzyme that transforms chorismate into prephenate. Prephenate dehydratase converts prephenate into arogenate. Finally, by the activities of arogenate dehydratase and transaminase, arogenate is converted to phenylalanine (Dawson 1986; Herrmann and Weaver 1999). Phenylalanine exhibits antioxidant activities and regulates the expression level of NRF2 and its target antioxidant enzymes, such as SOD and GP_x_, in grass carp, improving its intestinal immune status shellfish (Feng et al. 2017b).

### Threonine

Threonine (**7**) (Table 1) is an essential amino acid. Its biosynthetic pathway begins with the condensation of aspartate and ATP (adenosine triphosphate) to form β-aspartyl phosphate, a process catalyzed by aspartate kinase. The enzymes homoserine dehydrogenase and aspartate-semialdehyde dehydrogenase then work together to convert β-aspartyl phosphate to homoserine through a sequence of activities. Under the influence of homoserine kinase, homoserine is phosphorylated to produce homoserine phosphate. Next, threonine synthase converts homoserine phosphate to threonine (Raïs et al. 2001). Its antioxidant boosts the immune system’s ability to fight free radicals, protecting the body's equilibrium (Ji et al. 2019; Egbujor et al. 2020a). Dietary threonine elevates mRNA levels of NRF2 and its dependent antioxidant enzymes and improves growth performance and muscle growth (Zhao et al. 2020).

### Tryptophan

Tryptophan (**8**) (Table 1) is an indole containing essential α-amino acid, which serves as a precursor to melatonin, serotonin, and vitamin B3 (Slominski et al. 2002). Its biosynthesis follows the shikimate pathway. The conversion of erythrose-4-phosphate and phosphoenolpyruvate to chorismate initiates the shikimate pathway, a major metabolic route. 3-deoxy-d-arabino-heptulosonate-7-phosphate synthase (DAHPS) is the enzyme that catalyzes this process. Anthranilate synthase then converts chorismate to anthranilate through this process. Indole-3-glycerol phosphate (IND) is created when anthranilate and phosphoribosyl pyrophosphate (PRPP) are mixed. Phenol phosphoribosyltransferase is the catalyst for this process which finally leads to tryptophan production (Radwanski and Last 1995). Industrially, tryptophan biosynthesis involves the fermentation of serine and indole using bacteria (Ikeda 2003). Tryptophan enhances antioxidant capacity in the placenta (Xu et al. 2018) and protects hepatocytes by NRF2-gene induction amongst multiple pathways (Kimura and Watanabe 2016). Wen et al. (Wen et al. 2014) reported that supplementation of dietary tryptophan upregulates NRF2 and its target antioxidant genes, thereby improving the antioxidant status of grass carp. The ability of optimal dietary tryptophan to improve grass carp's flesh quality and muscle content has been linked to its anti-oxidative action via the activation of NRF2 and target of rapamycin (TOR) signaling factors (Jiang et al. 2016c).

### Valine

Valine (**9**) (Table 1) is obtained from dairy sources such as dairy products, meat, beans, and legumes. Threonine is an amino acid that is produced via the aspartate pathway and serves as a precursor for the biosynthesis of valine. α-Ketobutyrate is produced when threonine is deaminated by threonine dehydrogenase. Then, 2-acetolactate is formed when α-ketobutyrate and pyruvate combine, a process that is mediated by acetohydroxy acid synthase (AHAS). Following 2-acetolactate, the enzyme acetohydroxy acid isomeroreductase (also called ketol-acid reductoisomerase, KARI) transforms it into 2,3-dihydroxy-isovalerate. Dihydroxyacid dehydratase dehydrates 2,3-dihydroxy-isovalerate to produce α-ketoisovalerate. A transaminase enzyme catalyzes the transamination of α-ketoisovalerate to produce valine. An amino group is moved from glutamate to α-ketoisovalerate in this phase (Oldiges et al. 2014). It exhibits antioxidant properties (Cojocaru et al. 2014). Dietary valine supplementation improves the antioxidant status of young grass carp by elevating the mRNA levels of NRF2-dependent antioxidant enzymes, including SOD1, CAT, and GP_x_, via the activation of NRF2 signaling, amongst other pathways (Luo et al. 2017). Dietary valine significantly activates NRF2 in juvenile hybrid grouper and improves its antioxidant status and growth (Zhou et al. 2021).

### Taurine

Taurine (**10**) (Table 1) is a sulfur-containing essential amino acid. In the biosynthesis of taurine, first, methionine must be converted to methionine sulfoximine (MSO), an enzyme that is catalyzed by methionine sulfoximine transaminase. The enzyme methionine sulfoximine transaminase then converts MSO to sulfinoalanine. The succeeding process, which is performed by cysteine synthase, converts sulfinoalanine into cysteine. The enzyme cysteine dioxygenase proceeds with the oxidation of cysteine to generate cysteine sulfinic acid. The last stage in the production of taurine is the transformation of cysteine sulfinic acid into taurine which is catalyzed by taurine synthase or cysteine sulfinic acid decarboxylase (Husna et al. 2018). Age-related health issues can be prevented in aging mice, worms, and monkeys by supplementing with taurine, which has been proven to counteract the fall in taurine levels as animals age. Additionally, taurine's ability to support mitochondrial homeostasis may be a factor in how it affects health (Singh et al. 2023). (Yang et al. 2017) reported that taurine reduces ROS production and mitigates ionizing radiation-induced GC-2 cell damage by activating the NRF2/HO-1 pathway. Similarly, Agca and co-workers (Agca et al. 2014) reported that taurine activates the NRF2/HO-1 signaling pathway and attenuates the severity of oxidative stress by improving NRF2 and HO-1 expression levels in diabetic rats.

### Role of non-essential amino acids in NRF2 activation

#### Serine

Serine (**11**) (Table 2) is a non-essential amino acid synthesized by the oxidation of 3-phosphoglycerate (3-PG) to 3-phosphohydroxypyruvate (3-PHP), which undergoes transamination and hydrolyzation. In the biosynthesis of serine, the initial substrate is 3-PG, which is an intermediate in glycolysis. 3-PG is converted to 3-PHP by the enzyme phosphoglycerate dehydrogenase (PHGDH). The first step in the serine biosynthesis pathway is this reaction. In the second phase, the enzyme phosphoserine aminotransferase (PSAT1) transforms 3-PHP into phosphoserine. Finally, phosphoserine phosphatase (PSPH) breaks down phosphorine to produce serine (Nelson et al. 2021). Serine exhibits significant antioxidant activities (Maralani et al. 2012; Egbujor et al. 2019). (Chen et al. 2021) reported that serine supplementation could represent a nutritional strategy for improving skeletal muscle functions in doxorubicin-exposed patients. It has been found to attenuate doxorubicin-induced oxidative stress and its concomitant effects in the skeletal muscle of mice via the activation of the NRF2/constitutive-androstane-receptor (CAR) signaling pathway. Serine supplementation also maintains redox balance and attenuates oxidative stress via glutathione synthesis and activation of NRF2 signaling in intestinal porcine epithelial cells (IPEC-J2) (He et al. 2020).

**Table 2:** Non-essential amino acids and NRF2 activation

S/N	Molecular structure	Biological activity	Effective concentration	Study model	NRF2 target genes	Disease of interest	Reference
11	Serine	Antioxidant	1.5 g/kg	Mice	SOD CAT	Oxidative stress	(Chen et al. 2021)
		Antioxidant	1.2 mM	C57BL/6 J Mice	GPx	Oxidative stress	(He et al. 2020)
12	Arginine	Antioxidant	25–100 mg/100 g BW	Rats	SOD CAT GPx	Oxidative stress	(Liang et al. 2018b)
		Antioxidant	2.25%	Rats	CAT GPx	Oxidative stress	(Ramprasath et al. 2012)
		Antioxidant	0.5–30 mM	C2C12 myotube cells	SOD CAT GPx	Oxidative stress	(Zhao et al. 2021b)
		Antioxidant	155 mmol/kg	Ewes	SOD CAT GPx	Oxidative stress	(Ma et al. 2022)
		Antioxidant	300 µg/kg/min	Rats	SOD HO-1	Renal ischemia/reperfusion injury	(Tong and Zhou 2017)
		Antioxidant	100 µM	Ovine intestinal epithelial cells	NQO1 CAT SOD	Oxidative stress	(Zhang et al. 2019)
		Antioxidant	100 mg/kg	Rats	HO-1	Ototoxicity	(Estfanous et al. 2020)
13	Glutamine	Antioxidant Anticancer	2 mM	HT29 and HCT116 cells	GPx	Colon cancer	(Polat et al. 2021)
		Antioxidant	4%	Rats	GCLM	Oxidative stress	(Venoji et al. 2015)
		Antioxidant	1 g/kg	Rats	HO-1 SOD GPx	Intestinal ischemia/reperfusion injury	(Wang et al. 2015)
		Antioxidant	0.5–1.5%	Broilers	SOD CAT GPx	Oxidative stress	(Hu et al. 2020)
		Antioxidant	–	Rats	HO-1, NQO1	Traumatic brain injury	(Shukai et al. 2019)
		Antioxidant	8 g/kg	Grass carp	SOD GPx	Oxidative injury	(Jiang et al. 2016a)
14	Glycine	Antioxidant	1% (w/v)	Rats	SOD CAT NQO1 HO-1	Diabetes	(Wang et al. 2019b)
		Antioxidant	1000 mg	Mice	NQO1 HO-1	Acute lung injury	(Zhang et al. 2020)
**15**	Homocysteine	Antioxidant	50 µM–1 mM	Muller glial cells	NQO1 CAT SOD2 GPx	Oxidative stress	(Navneet et al. 2019)
**16**	N-Acetylcysteine	Antioxidant	600 mg	Infertile men with asthenoteratozoospermia	CAT SOD GPx	Oxidative stress	(Jannatifar et al. 2020)
**17**	Carbocysteine	Antioxidant	10^–4^ M	Bronchial epithelial cells (16-HBE)	HO-1	Chronic obstructive pulmonary disease (COPD)	(Pace et al. 2013)

#### Arginine

Arginine (**12**) (Table 2) serves as a precursor of nitric oxide. It is commonly obtained by the hydrolysis of gelatin or commercially by fermentation. The biosynthesis of arginine begins with the conversion of ornithine, a precursor amino acid derived from glutamate. The ornithine transcarbamylase (OTC) enzyme is responsible for catalyzing this reaction. After that, ornithine reacts with carbamoyl phosphate, a substance produced during the urea cycle to form citrulline and this process is catalyzed by ornithine carbamoyltransferase (OT). Citrulline and aspartate react to form argininosuccinate under the catalysis of argininosuccinate synthase (ASS). The argininosuccinate is broken down into arginine and fumarate, a process catalyzed by argininosuccinate lyase (ASL) (Urbano-Gámez et al. 2020). It exhibits antioxidant activities and attenuates oxidative stress (Egbuonu 2022). l-Arginine inhibits oxidative stress and enhances antioxidant defenses by stimulating glutathione synthesis and activating the NRF2–KEAP1 signaling pathway (Liang et al. 2018b). l-Arginine supplementation improves antioxidant responses in hyperglycemic rats by attenuating cardiac left ventricular oxidative stress via the activation of NRF2 and eNOS signaling and upregulation of their target genes (Ramprasath et al. 2012). l-Arginine, by activating SIRT-AKT-NRF2 among other pathways, attenuates lipopolysaccharide (LPS)-induced oxidative stress and cell death in myotube cells (Zhao et al. 2021b). Arginine supplementation significantly improves the ovarian antioxidant capacity of ewes during the luteal phase via the activation of NRF2–KEAP1 signaling pathway and elevation of the NRF2 target antioxidant enzymes such as HO-1, SOD, and GPx (Ma et al. 2022). Arginine also decreases ROS production and expression of NF-ĸB and exerts therapeutic effects against renal ischemia–reperfusion injury by activating rats' NRF2/HO-1 pathway (Tong and Zhou 2017). Similarly, arginine activates NRF2 and upregulates the expression of the phase II metabolizing and antioxidative enzymes, thereby protecting ovine intestinal epithelial cells from LPS-induced cell death and oxidative damage (Zhang et al. 2019). It also attenuates cisplatin–induced ototoxicity via the activation of the NRF2/HO-1 pathway among others in rats (Estfanous et al. 2020).

#### Glutamine

Glutamine (**13**) (Table 2) is the most abundant amino acid in the body. It is synthesized by glutamine synthetase from glutamate and ammonia. It is also produced industrially with mutants of *Brevibacterium flavum*. Glutamate serves as the common precursor for glutamine biosynthesis. Glutamate is an amino acid derived from several sources, including the transamination of other amino acids. The enzyme glutamate dehydrogenase is responsible for the conversion of ammonia and alpha-ketoglutarate into glutamate. Glutamine synthetase (GS) is the primary enzyme involved in the production of glutamine from glutamate. It facilitates the ATP-dependent condensation of ammonia and glutamate to produce glutamine (Newsholme et al. 2003; Elvers and Bellussi 2011). Glutamine exhibits significant antioxidant activity (Nemati et al. 2019). (Polat et al. 2021) reported that the function and expression of glucose 6-phosphate dehydrogenase is modulated by glutamine via the activation of NRF2 in colon cancer cells. Glutamine supplementation enhances glutathione levels via the regulation of NRF2. It upregulates NRF2 and induces the expression of glutathione synthetase and glutathione levels in the villus-crypt of the intestine (Venoji et al. 2015). Glutamine also attenuates intestinal ischemia–reperfusion injury via the activation of the NRF2/ARE signaling pathway in rats. It also raises the amounts of mRNA and protein in NRF2 target genes such as HO-1, SOD, and GPx (Wang et al. 2015). Additionally glutamine reduces heat stress-related oxidative damage by activating the NRF2–KEAP1 signaling pathway in the broiler's thigh muscle (Hu et al. 2020). Glutamine supplementation in rats inhibits traumatic brain injury (TBI)-induced oxidative stress, reduces neuron apoptosis, and promotes autophagy via the activation of NRF2 and elevation of its antioxidant enzymes (Shukai et al. 2019). Glutamate is also attenuates copper-mediated oxidative damage and improves antioxidant defenses via NRF2 modulation in grass carp intestines (Jiang et al. 2016a).

#### Glycine

Glycine (**14**) (Table 2) is the simplest stable amino acid and the most abundant in collagen, acting as an inhibitory neurotransmitter (Razak et al. 2017). The biosynthesis of glycine begins with the conversion of serine. The enzyme serine transhydroxymethylase, or serine hydroxymethyltransferase (SHMT), catalyzes a reversible reaction with serine. In this process, serine gives tetrahydrofolate (THF) a one-carbon unit, which forms 5,10-methylene tetrahydrofolate and glycine (Keys 1980). Glycine inhibits the formation of advanced glycation end-products and renal oxidative damage by increasing the function of glyoxalase-1 via the activation of NRF2 in the kidney of streptozotocin-induced diabetic rats (Wang et al. 2019b). Inhibition of LPS-mediated acute lung damage by glycine pretreatment in mice is achieved by regulating NRF2 and NLRP3 inflammasome signaling (Zhang et al. 2020).

#### Cysteine

Cysteine (**15**) (Table 2) is a non-essential amino acid. Homocysteine an intermediate in methionine cycle is the precursor for cysteine biosynthesis. Methionine adenosyltransferase catalyzes the conversion of methionine, an essential amino acid, to S-adenosylmethionine (SAM). The next step involves a process called transmethylation or trans-sulfuration that turns SAM into homocysteine. Cystathionine is then formed by the interaction of homocysteine with serine, which is mediated by cystathionine beta-synthase (CBS). The enzyme cystathionine gamma-lyase then transforms cystathionine into cysteine (CSE) (Rehman et al. 2019). Cysteine derivatives have been shown to activate NRF2 signaling pathways. Homocysteine lowers oxidative stress and ROS levels by triggering the NRF2 pathway in retinal Muller glial cells and upregulating NRF2 expression (Navneet et al. 2019). It has been discovered that oral *N*-acetyl cysteine (NAC) (**16**) (Table 2) supplementation increases the expression of NRF2 genes and antioxidant enzymes. It improved the quality of sperm in asthenoteratozoospermic men by protecting them from oxidative damage through NRF2 activation (Jannatifar et al. 2020). In addition, carbocysteine (**17**) (Table 2) has been shown to boost HO-1, GSH, and NRF2 expression and activity in bronchial epithelial cells that have been induced by cigarette smoke extract (Pace et al. 2013).

### Potential negative effects of amino acid-mediated NRF2 Activation

While several studies have suggested that amino acid-mediated NRF2 activation exerts positive effects on cells, studies with negative results may have been underreported. It is worth to note that not all amino acid-related NRF2-regulated processes are favorable. Although it is well established that NRF2-mediated cysteine accumulation enhances the production of other cysteine-derived metabolites, it is unclear if this control of cysteine metabolism by NRF2 also significantly raises GSH levels after NRF2 activation (DeBlasi and DeNicola 2020). The stabilization of cysteine dioxygenase 1 (CDO1) and enhanced entrance of cysteine into the taurine production pathway led to cysteine buildup mediated by NRF2, which was a metabolic vulnerability in non-small cell lung cancer (NSCLC) cells (Kang et al. 2019). This caused excessive cystine reduction, which in turn hindered NSCLC growth and antioxidant activity, resulting in the generation of wasteful and toxic products and the depletion of NADPH. The prolonged activation of the NRF2 protein in many cancers confers resistance to chemotherapy and radiation treatment on tumor cells. Therefore, it has been demonstrated that inhibiting aberrant NRF2 activity in malignancies lowers resistance in disease models (Srivastava et al. 2022). A general dependence on exogenous non-essential amino acids has been observed in cancers with strong antioxidant capacities. This dependence is caused by the NRF2-dependent glutamate secretion via system xc- (XCT), which restricts intracellular glutamate pools necessary for non-essential amino acids synthesis. Interestingly, tumors may be made more sensitive to the dietary limitation of non-essential amino acids without any changes to the Keap1/NRF2 pathway by reducing endogenous glutamate levels by glutaminase inhibition (LeBoeuf et al. 2020). Thus, limiting external inputs of non-essential amino acids could be a metabolic strategy to therapeutically target malignancies with genetic or pharmacologic activation of the NRF2 antioxidant response pathway. In addition, several amino acids themselves do not inherently cause oxidative stress, however, some of them can generate ROS through metabolic processes. For instance, phenylalanine depletes antioxidant defense and elevates oxidative stress by increasing the production of reactive oxygen–nitrogen species (Kumru et al. 2019). ROS can also be produced as byproducts of the breakdown of amino acids through mechanisms such as amino acid catabolism or the synthesis of certain neurotransmitters (Forrester et al. 2018). Reactive intermediates can be formed by oxidizing aromatic amino acids such as phenylalanine and tyrosine. These intermediates could be a part of oxidative stress under specific circumstances. It is usually not a significant generator of ROS in physiological settings and concentrations, though (Fitzpatrick 2003). Different metabolites that result from tryptophan metabolism may possess pro-oxidant characteristics. A tryptophan-catabolism-related enzyme called indoleamine 2,3-dioxygenase (IDO) produces kynurenine, whose metabolism can cause reactive oxygen species (ROS) (Sadok and Jędruchniewicz 2023). Nitric oxide, which is something that arginine may help produce, has the potential to exacerbate oxidative stress in some situations (Pizzino et al. 2017). Despite its vital physiological functions, NO can cause oxidative damage when it is produced excessively or reacts with other ROS.

## Conclusion

NRF2 is an essential transcription factor for the oxidant stress response, but it also plays a crucial role in the control of inflammation, immunological responses, metabolism, and mitochondrial physiology. The activating stimulus, the cellular environment, the presence of binding partners, interactions with other transcription factors, all influence NRF2-mediated target gene expression. Several studies have shown that amino acids and their metabolites are also involved in several cellular processes including NRF2 activation and consequently antioxidant and anti-inflammatory functions. Also, amino acids facilitate the synthesis and maintenance of essential antioxidants like glutathione, which help scavenge harmful free radicals and protect cells from oxidative stress. Although lot of research has been focused on the role of different amino acids, however, it is necessary to understand role of the amino acid in this cross-regulation for the designing of better therapeutic approaches. Only studies that have looked into the interactions between amino acids and NRF2 have been included in this review. No study found a lack of interaction, but we should keep in mind that unfavorable findings might not have been reported and that the other amino acids might not have been examined for NRF2 interaction yet. The findings obtained regarding NRF2 activation and non-essential amino acids has been obtained in vitro and animal models. The rats and mice were employed as established animal models. Otherwise, the conclusions regarding essential amino acids are based on multiple fish-based studies. In biomedical research, fish have become a popular substitute model organism for performing toxicological and pharmacological experiments. Two common models used extensively in pharmaceutical research are zebrafish and medaka (Supriya et al. 2022). Unfortunately, other fish species that are used in zootechnical research rather than medical research have been used in the reported studies. However, as is the case with methionine and tryptophan, the outcomes observed in these fish models are validated in more conventional animal models (rats or mice). It is evident, for essential amino acids, that additional data from well-established preclinical models are required in order to convert preclinical discoveries into clinical practice. The glutamine data are consistent with its usage as a supplement to boost muscle development and recovery, enhance exercise performance, foster immunological function, and treat some gut-related diseases like inflammatory bowel disease or leaky gut syndrome. The availability and distribution of glutamine supplements have been quite problematic, and they have an affinity for gut and renal tissue (Buckingham 2020). Studies considered for this review did provide outcomes in renal and gastrointestinal illness animal models. The presented information about cysteine derivatives also relates to the various medicinal uses. Indeed, *N*-acetylcysteine and carbocysteine are known for their mucolytic and antioxidant properties. Moreover, *N*-acetylcysteine is used as an antidote for acetaminophen (paracetamol) overdose, as it helps replenish glutathione (Buckingham 2020). It is worth noting that the paucity of information from human studies on the supplementation of amino acids and NRF2 function is the central issue. Clinical research on derivatives of glutamine and cysteine has gathered strong scientific evidence to support their use under specific circumstances. However, there are a number of confounding factors that make conducting these studies extremely difficult. The prospective, randomized, controlled scientific trial is considered the “gold standard” for examining the effects of amino acids under standardized conditions. However, the influence of amino acid supplementation on NRF2 function may differ depending on factors like general health, dietary habits, and specific nutritional requirements. This review’s data collection aims to encourage research on the NRF2 pathway in clinical studies involving amino acids, particularly those for which promising preclinical data are available. Furthermore, it is necessary to establish the role of amino acids as possible therapeutics. It is important to close knowledge gaps in oxidative stress and inflammation-related diseases to suggest potential treatments for conditions where oxidative stress plays a major role.

## Data Availability

Data sharing is not applicable to this article as no new data were created or analyzed in this study.
